# 2,4-Dichlorophenoxyacetic Thiosemicarbazides as a New Class of Compounds against Stomach Cancer Potentially Intercalating with DNA †

**DOI:** 10.3390/biom10020296

**Published:** 2020-02-13

**Authors:** Monika Pitucha, Agnieszka Korga-Plewko, Pawel Kozyra, Magdalena Iwan, Agnieszka A. Kaczor

**Affiliations:** 1 Independent Radiopharmacy Unit, Department of Organic Chemistry, Faculty of Pharmacy, Medical University of Lublin, PL-20093 Lublin, Poland; 2 Independent Medical Biology Unit, Faculty of Pharmacy, Medical University of Lublin, PL-20093 Lublin, Poland; agnieszkakorga@umlub.pl (A.K.-P.);; 3 Student Research Group, Independent Radiopharmacy Unit, Faculty of Pharmacy, Medical University of Lublin, PL-20093 Lublin, Poland; 4 Department of Synthesis and Chemical Technology of Pharmaceutical Substances, Faculty of Pharmacy, Medical University of Lublin, PL-20093 Lublin, Poland; agnieszka.kaczor@umlub.pl; 5 School of Pharmacy, University of Eastern Finland, Yliopistonranta 1, P.O. Box 1627, FI-70211 Kuopio, Finland

**Keywords:** thiosemicarbazide, cytotoxicity, cell cycle, DNA, DNA intercalators

## Abstract

Thiosemicarbazide is a useful structural moiety that has the biological potential. Optimization of this structure can result in groundbreaking discovery of a new class of therapeutic agents. In the light of this, 1-(2,4-dichlorophenoxy)acetyl-4-(1-naphthyl)thiosemicarbazide (**1**) and 1,4-bis[(2,4-dichlorophenoxy)acetylthiosemicarbazide]phenyl (**2**) were synthesized and characterized by spectroscopic method. Cytotoxicity of obtained compounds was evaluated on MKN74 gastric cancer cell line and human skin fibroblast BJ based on methylthiazolyldiphenyl-tetrazolium bromide (MTT) test. The apoptosis/necrosis and cell cycle analysis were conducted using image cytometry. Additionally, in DNA of treated cells, abasic sites (AP) and double strands breaks (DSB) presence were measured. Intercalating properties of active compounds were evaluated using the UV–spectroscopic method. Among newly synthesized derivatives, compound **2** showed toxic effects on gastric cancer cells with simultaneous lack of toxicity to normal fibroblasts. Cell cycle analysis revealed that both compounds influence cell division mainly at the stage of replication. Simultaneously with DNA synthesis disorders, DNA damages like AP-sites and DSBs were observed. Spectroscopic studies revealed possible DNA intercalating properties of tested compounds. Obtained results indicate that the newly synthesized thiosemicarbazide derivatives are a promising group of compounds with potential anticancer activity resulted from interactions with DNA and cell cycle interrupt.

## 1. Introduction

Cancer belongs to the main reasons for death worldwide in countries of all income levels [1]. According to WHO, cancer is the second leading cause of death globally and is estimated to account for 9.6 million death in 2018. Globally, about 1 in 6 deaths is due to cancer. Lung, prostate, colorectal, stomach, and liver cancer are the most common types of cancer in men, while breast, colorectal, lung, cervix, and thyroid cancer are the most common among women [2]. Among various types of cancer, gastric cancer is the second most common reason for cancer-related deaths worldwide [3].

Surgery and radiotherapy are the most efficient and valuable methods for local and non-metastatic cancer treatment, but they are not effective when cancer has spread throughout the organism [4]. The application of anticancer drugs, i.e., chemotherapy, hormone, and biological therapies, is the current method of choice for the treatment of metastatic cancers since they are capable of reaching each part of the body via the bloodstream [4]. The currently available anticancer drugs can be divided into cytotoxic-based drugs and target-based drugs. The cytotoxic compounds are able to rapidly kill dividing cells by targeting components of the mitotic and/or DNA replication pathways while target-based drugs block the cancer growth by interactions with molecular targets involved in cancer progression [5]. Novel drugs to treat cancer are still developed [6,7,8,9] to meet the need of alarming cancer epidemiology.

Our research focuses for a long time on searching for novel compounds with anticancer activity [10,11,12,13]. In particular, we focus on thiosemicarbazides as a privileged scaffold in medicinal chemistry, displaying a wide range of activities, i.e., antibacterial, antifungal, antioxidant [14,15,16,17,18], and anticancer activity [17,18,19]. Among a series of different thiosemicarbazides derivatives studied against human gastric cancer cell line MKN74, we selected two most promising based on IC_50_ values. Here we present synthesis and characterization of these two compounds, cell cycle analysis, oxidative DNA damage, and spectrophotometry study on the interactions of the compounds with ds-DNA as well as molecular docking results on the interactions of the compounds with DNA.

## 2. Materials and Methods

All chemicals used for the synthesis were purchased from Sigma-Aldrich (Sigma-Aldrich, St. Louis, MO, USA), Alfa Aesar (Haverhill, MA, USA), and POCH (Polish Chemical Reagents, Gliwice, Poland) companies. Melting points were determined using Fisher–Johns block. The ^1^H and ^l3^C NMR spectra were recorded on a Bruker Avance 600 spectrometer (Bruker BioSpin GmbH, Rheinstetten, Germany). The attenuated total reflectance ATR-IR spectra were recorded on the Thermo Scientific Nicolet 6700 FTIR spectrophotometer (Thermofisher, Waltham, Massachusetts, USA). The mass measurements were made using an Agilent Technologies liquid chromatograph 1290 coupled to an Agilent Technologies 6550 iFunnel Q-TOF LC/MS (Agilent Technologies, Santa Clara, CA, USA) [20]. Compound 1 was first obtained by Gupta et al. [21].

### 2.1. General Procedure for the Synthesis of 1,4-Disubstituted Thiosemicarbazide Derivatives (**1**,**2**)

0.5 g of 2,4-Dichlorophenoxyacetic acid hydrazide was mixed with appropriate isothiocyanate (1-naphthylisothiocyanate or 1,4-phenylenediisothiocyanate) in 15 mL methanol solution. Next, the mixture was heated at reflux temperature for 2h. After cooling, the target substance was filtered off and crystallized from methanol.

#### 2.1.1. 1-(2,4-Dichlorophenoxy)acetyl-4-(1-naphtyl)thiosemicarbazide (**1**) [21]

Yield: 80 %, m.p. 161-162 °C. IR cm^−1^: 3307 (NH), 3183 (NH), 3057 (NH), 1661 (C=O). ^1^H NMR (DMSO-d_6_) δ ppm: 4.86 (s, 2H), 7.16-7.62 (m, 7H, CH_ar_), 7.87-7.97 (m, 3H, CH_ar_), 9.78 (s, 1H, NH), 9.94 (s, 1H, NH), 10.41 (s, 1H, NH). ^13^C NMR (DMSO-d_6_) δ ppm: 67.1; 115.9; 122.9; 124.1; 125.5; 125.9; 126.4; 126.5; 127.0; 127.5; 128.3; 129.3; 131.1; 134.2; 135.9; 153.1; 167.1; 170.6; 182.9. LC-QTOF MS (*m/z*): calculated monoisotopic mass: 419.0262, measured monoisotopic mass: 419.0266 (Figure S1).

#### 2.1.2. 1-[[2-(2,4-Dichlorophenoxy)acetyl]amino]-3-[4-[[[2-(2,4-dichlorophenoxy)acetyl]amino] carbamoylamino]phenyl]thiourea (2)

Yield: 77 %, 158-159 °C. IR cm^−1^: 3315 (NH), 3165 (NH), 1693 (C=O).^1^H NMR (DMSO-d_6_) δ ppm: 4.80 (s, 4H, 2xCH_2_), 7.14-7.36 (m, 2H, CH_ar_), 7.35-7.43 (m, 6H, CH_ar_), 7.55-7.60 (m, 2H, CH_ar_), 9.68-9.86 (m, 4H, 4NH), 10.26 (s, 2H, 2NH). ^13^C NMR (DMSO-d_6_): 67.0; 115.5; 115.9; 122.5; 122.8; 125.0; 125.7; 126.2; 126.8; 128.1; 128.3; 129.8; 133.6; 136.5; 139.0; 152.0; 153.0; 167.3; 170.7; 182.2. LC-QTOF MS (*m/z*): calculated monoisotopic mass: 659.9742, measured monoisotopic mass: 659.9736 (Figure S2).

### 2.2. Cell Culturing

The human gastric cancer cell line MKN74 (Japanese Cancer Research Resources Bank, JCRB, Tokyo, Japan) and human skin fibroblast BJ (ATCC, Manassas, Wirginia, USA) were cultured in RPMI and Eagle’s Minimum Essential Medium (EMEM) (Corning, New York, NY, USA) respectively, supplemented with 10 % fetal bovine serum (Corning, New York, NY, USA) and a set of antibiotics: penicillin (100 units) and streptomycin (100 µg/mL) (Corning, New York, NY, USA). Cells were incubated at 37 °C with 5% CO_2_ in air atmosphere. The cell morphology was analyzed under a phase-contrast microscope Nikon Eclipse Ti using NIS-Elements Imaging Software (Nikon, Tokyo, Japan). The authenticity of tested cell lines was verified by short tandem repeat (STR) genotyping in the Department of Forensic Medicine (Medical University of Lublin, Lublin, Poland).

### 2.3. The Cytotoxicity Analysis

The cytotoxicity of tested compounds was evaluated using the MTT (methylthiazolyldiphenyl-tetrazolium bromide) test. The test is based on the living cells’ ability to reduce orange tetrazolium salt to water-insoluble purple formazan crystals. The cells were seeded into 96-well plates: MKN74 in the concentration of 1.5 × 10^5^ cells/mL and BJ in the concentration of 1 × 10^5^ cells/mL. The tested compounds (in the final concentration of 10, 125, and 250 µM) were added when 70%–80% of confluence was achieved. The MTT-3-(4,5-dimethylthiazol-2-yl)-2,5diphenyltetrazolium bromide (Thermofisher, Waltham, Massachusetts, USA) solution (4.0 mg/mL) was added to the culture 24 h after chemicals. After 4 h of incubation, the medium with MTT was removed, and the formed crystals were dissolved in DMSO (dimethyl sulfoxide, 200 mL/well, POCH, Poland). The solution absorbency was measured at 540 nm using PowerWave microplate spectrophotometer (BioTek Instruments, USA). Each assay was conducted three times and was measured in triplicates. IC_50_ values were determined using the AAT Bioquest IC_50_ calculator [22].

### 2.4. Cell Cycle Analysis

Cell cycle analysis was conducted using NucleoCounter NC-3000 (ChemoMetec, Lillerød, Denmark) according to manufacturer instructions. The MKN74 cells were seeded into 6-well plates at a concentration of 1.5 × 10^5^ cells/mL and the tested compounds were added when 70%–80% of confluence was achieved. After 24 h of incubation with tested compounds, the cells were collected and analyzed according to the manufacturer’s protocol-2-step Cell Cycle Assay (ChemoMetec, Lillerød, Denmark). The culture medium was removed and cells were washed with PBS and resuspended in Solution 10 supplemented with DAPI (4′,6-Diamidine-2′-phenylindole dihydrochloride) 10 μg/mL. Then, the cells were incubated at 37 °C for 5 minutes, and Solution 11 was added. Thirty microliters of the cell suspension was loaded into NC-Slide and read in NucleoCounter. Each experiment was conducted three times with measurement in triplicate.

### 2.5. Determination of DNA Oxidative Damage

The oxidative DNA damage was evaluated by measuring the number of abasic sites (AP sites) that are one of the major types of damage generated by reactive oxygen species (ROS). The cells were seeded into 6-well plates in a concentration of 1.5 × 10^5^ cells/mL. The tested compounds (in a final concentration of 10, 125, and 250 µM) were added when 70%–80% of confluence was achieved. After 24-h incubation, the DNA was isolated with the Syngen DNA Mini Kit (Syngen, Poland) according to the manufacturer’s protocol. The concentration of the genomic DNA was measured using the MaestroNano Micro-Volume Spectrophotometer (Maestrogen Inc., Hsinchu, Taiwan) and adjusted to 100 μg/mL in the TE buffer. The number of abasic sites (AP sites) was evaluated with the DNA Damage Quantification Kit (Dojindo, Kumamoto, Japan) according to the manufacturer’s instructions. The method is based on a specific reaction of an aldehyde-reactive probe (ARP; N′-aminooxymethylcarbonylhydrazin-D-biotin) with an aldehyde group present on the open-ring form of AP sites. AP sites were tagged with biotin residues and were quantified using avidin-biotin assay followed by colorimetric detection of peroxidize-conjugated to the avidin at 650 nm using PowerWave™ microplate spectrophotometer (BioTek Instruments, Winooski, Vermont, USA). Each experiment was conducted three times with measurement in triplicate.

### 2.6. DNA Damage–Double Strand Breaks (DSB)

The cells were seeded into 96-well plates in a concentration of 1.5 × 10^5^ cells/mL. The tested compounds (in a final concentration of 10, 125, and 250 µM) were added when 70%–80% of confluence was achieved. After 24-h incubation, the DNA damage (DSB) was determined using the HCS DNA Damage Kit (Thermofisher, Waltham, Massachusetts, USA) according to the manufacturer’s instruction. Phosphorylation of H2A histones is a known response to DSBs formation specifically. The DSBs level was measured by specific antibody-based detection of phosphorylated H2AX (Ser139) in the nucleus. The fluorescence of secondary antibody was measured using the SpectraMax i3 Multi-Mode Platform (Molecular Devices, San Jose, California, USA). Each experiment was conducted three times with measurement in triplicate.

### 2.7. Spectrophotometry Study on the Interaction of the Compounds with ds-DNA

Calf thymus DNA (CT-DNA) was supplied by Sigma-Aldrich, (St. Louis, MO, USA). The stock solution of DNA was prepared by dissolving 0.25 mg of DNA in 1 mL of phosphate buffer (pH 7.4). The absorption was titrated by keeping the concentrations of CT DNA constant with varying concentrations of the tested compound—50, 40, 30, 20, and 10 µM. The absorption spectra were recorded in the wavelength range of 240–350 nm using PowerWave microplate spectrophotometer with 1-cm path length quartz cuvettes (BioTek Instruments, Winooski, Vermont, USA).

### 2.8. Statistical Analysis

The results were analyzed statistically in the STATISTICA vs. 12 application (StatSoft, Krakow, Poland). The data were calculated as mean ± SD. To compare more than two groups, one-way analysis of variance (ANOVA) and post hoc multiple comparisons with Tukey’s HSD test were used. All parameters were considered statistically significantly different if P values were less than 0.05.

### 2.9. Molecular Modeling

#### 2.9.1. Compound Preparation

Compounds **1** and **2** were modeled using LigPrep module [23] of Schrödinger suite of software, v. 2019-4 as previously reported [24,25,26,27,28]. To identify the protonation state, Epik module [29] of Schrödinger suite of software, v. 2019-4 was applied.

#### 2.9.2. Molecular Target

DNA fragment in complex with a threading intercalator 5-bromo-9-amino-*N*-ethy (diaminomethyl)acridine-4-carboxamide (PDB ID: 367D [30]) was used for molecular docking. DNA was prepared using respective tools of Schrödinger suite of software, v. 2019-4.

#### 2.9.3. Molecular Docking

Standard Precision (SP) method of Glide [31] from Schrödinger v. 2019-4 was used for molecular docking. The grid file was generated based on the co-crystallized ligand. 100 poses were obtained for each compound. The final poses were selected based on the Glide scoring function and visual inspection. Visualization of molecular modeling results was achieved with Maestro Release 2019.4 [32] and PyMol 2.0.4 [33] software.

## 3. Results and Discussion

**Figure 1:**
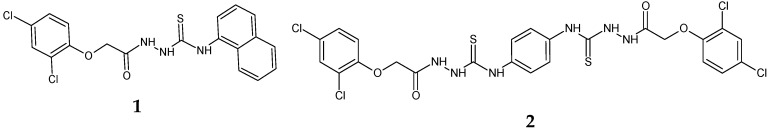
Structure of tested compounds.

The title compounds were obtained by reacting 2,4-dichlorophenoxyacetic hydrazide with 1-naphthylisothiocyanate (1) and 1,4-phenylene diisothiocyanate (2). The reactions were carried out in methanol in accordance with the procedures developed in our laboratory [12]. Structures of the obtained compounds (Figure 1) were determined by ^1^H and ^13^C NMR, IR, and MS.

**Figure 2:**
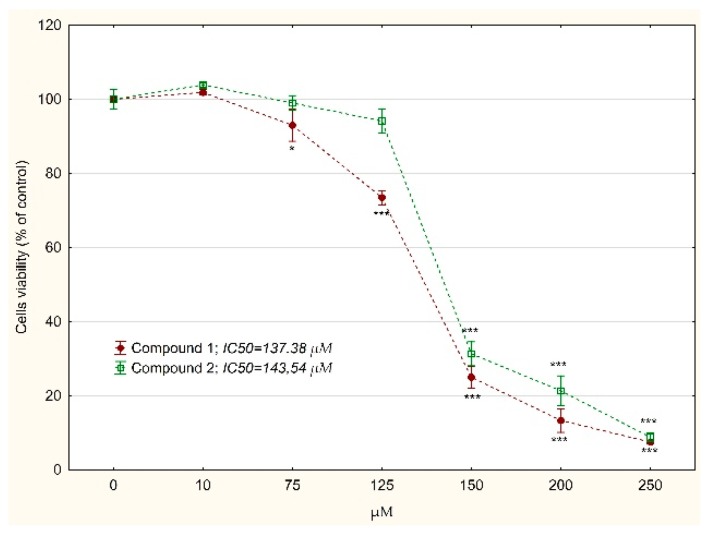
The relative MKN74 gastric cancer cells viability treated with compound 1 or 2 for 24 h determined by MTT assay. The results were calculated as % of control cultures’ viabilities which were averaged to define the 100%. Values were presented as mean ± SD derived from three independent experiments. * *p* < 0.05, *** *p* < 0.001 vs. control; (ANOVA followed by Tukey’s HSD post hoc analysis). IC_50_ value was determined using the AAT Bioquest IC_50_ calculator.

**Figure 3:**
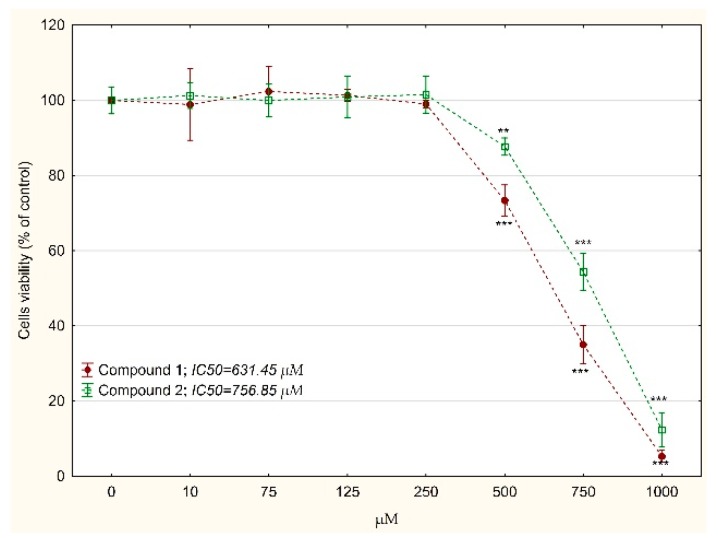
The relative BJ fibroblast cells viability treated with compound 1 or 2 for 24 h determined by MTT assay. The results were calculated as % of control cultures’ viabilities which were averaged to define the 100%. Values were presented as mean ± SD derived from three independent experiments. ** *p* < 0.01, *** *p* < 0.001 vs. control; (ANOVA followed by Tukey’s HSD post hoc analysis). IC_50_ value was determined using the AAT Bioquest IC_50_ calculator.

Cytotoxicity of tested compounds was evaluated by the MTT test. Both compounds revealed toxicity against MKN74 cells (Figure 2). Studies on normal human fibroblasts showed lower toxicity of the tested compounds: IC_50_ = 631.45 vs 137.38 µM for compound 1 and 756.85 vs. 143.54 µM for compound 2 (Figure 3).

**Figure 4:**
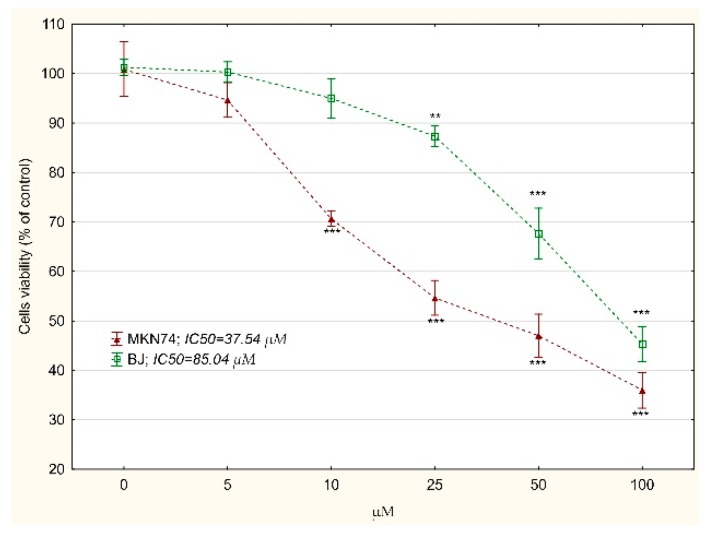
The relative MKN74 gastric cancer cells and BJ fibroblast viability treated with 5-fluorouracil for 24 h determined by MTT assay. The results were calculated as % of control cultures’ viabilities which were averaged to define the 100%. Values were presented as mean ± SD derived from three independent experiments. ** *p* < 0.01, *** *p* < 0.001 vs. control; (ANOVA followed by Tukey’s HSD post hoc analysis). IC_50_ value was determined using the AAT Bioquest IC_50_ calculator.

IC_50_ values for tested compounds were relatively high, as clinically useful drugs work at much lower concentrations. 5-fluorouracyl is the first-choice drug for the treatment of advanced gastric cancer but its effectiveness is limited by drug resistance [34]. The 5-fluorouracil toxicity toward MKN74 and BJ was determined. The obtained IC_50_ value in the case of cancer cells was clearly lower than the tested compounds: 37.54 vs. 631.45 or 756.85 µM (Figure 4). However, the ratio of IC_50_ values for normal vs. neoplastic cells was more beneficial for the tested compounds: 85.04/37.54 µM vs. 631.45/137.38 µM or 756.85/143.54 µM for compound 1 and 2, respectively. This observation justifies the further studies of new compounds that we obtained.

**Figure 5:**
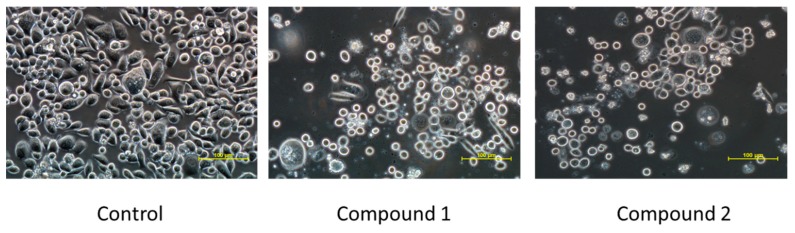
MKN74 cells morphology analyzed under a phase-contrast microscope Nikon Eclipse Ti. The cells were treated with compounds 1 or 2 (250 µM) for 24 h (magnification ×400).

The MTT test results for compounds 1 and 2 were verified by microscopic observations of cell morphology. The cells in control cultures revealed normal, epithelial-like morphology and were closely arranged and well adherent. Cells treated with compounds 1 and 2 for 24 h became round and had poor adherence especially in case of the highest concentrations of the compounds (Figure 5).

**Figure 6:**
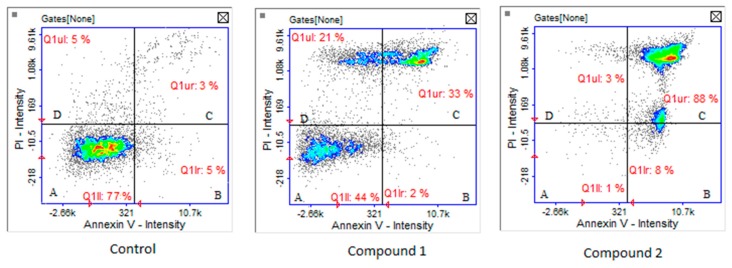
Cell apoptosis/necrosis of MKN74 cells, stained with Annexin V-FITC and PI for image cytometry, analyzed by NucleoCounter NC-3000. (**A**) Live, (**B**) early apoptotic, (**C**) late apoptotic, and (**D**) necrotic cells. The cells were treated with compounds 1 and 2 (250 µM) for 24 h.

Cytotoxicity was confirmed by apoptosis/necrosis analysis. After the use of compound **1** at a concentration of 250 µM, both late apoptotic and necrotic cells were observed. Compound **2** in the same concentration caused mainly apoptosis (88% of cells were in late apoptotic phase, Figure 6).

**Figure 7:**
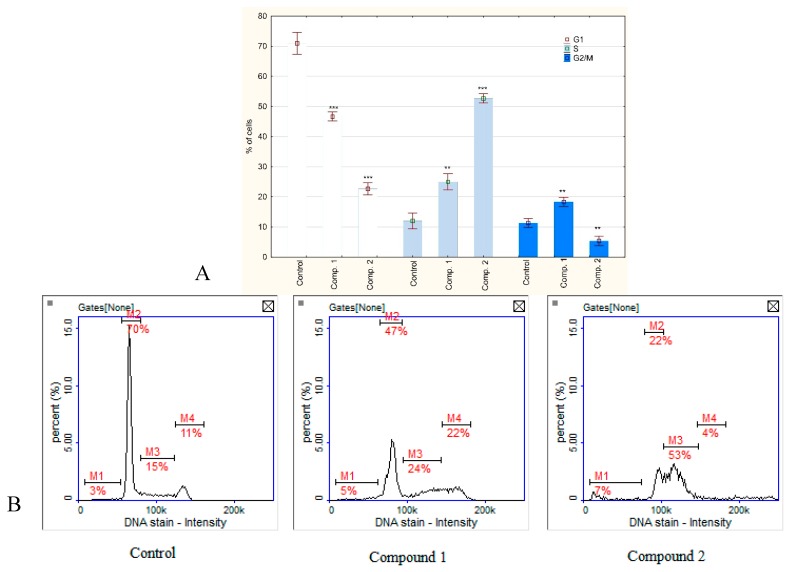
**(A**) Cell cycle analysis through DAPI (4′,6-Diamidine-2′-phenylindole dihydrochloride) staining and image cytometry by NucleoCounter NC-3000. The results were calculated as % of control values that were averaged to define the 100%. The MKN74 cells were treated with compounds **1** and **2** (250 µM) for 24 h. Values were presented as mean ± SD derived from three independent experiments. ** *p* < 0.01, *** *p* < 0.001 vs. control; (ANOVA followed by Tukey’s HSD post hoc analysis). (**B**) Representative histograms (M1-4: cell cycle phases subG1, G1, S, G2/M, respectively).

Cell cycle analysis by image cytometry revealed that both compounds arrested cell cycle. Compound **2** acted clearly in S phase while the action of compound **1** was more complex—an increase in the population of cells in either S-phase or G2 phase was observed. Due to the fact that DNA synthesis has been inhibited and consequently cell division, a decrease in cell population in G1 phase has been observed (Figure 7). These results indicate that both compounds influence cell division mainly at the stage of replication, although the mechanism of their action is not identical.

**Figure 8:**
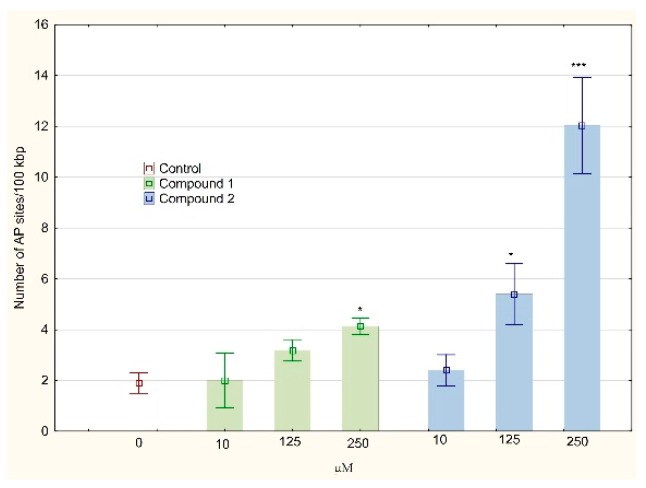
Abasic sites’ (AP) number per 100 kbp in DNA of MKN74 cells treated with compound 1 or 2 for 24 h. The AP site detection was based on a specific reaction of an aldehyde-reactive probe (ARP) with an aldehyde group present on the open-ring form of AP sites. AP sites were tagged with biotin residues and were quantified using avidin-biotin assay followed by colorimetric detection of peroxidase conjugated to the avidin at 650 nm. Values were presented as mean ± SD. * *p* < 0.05, *** *p* < 0.001 vs. control; (ANOVA followed by Tukey’s HSD post hoc analysis).

**Figure 9:**
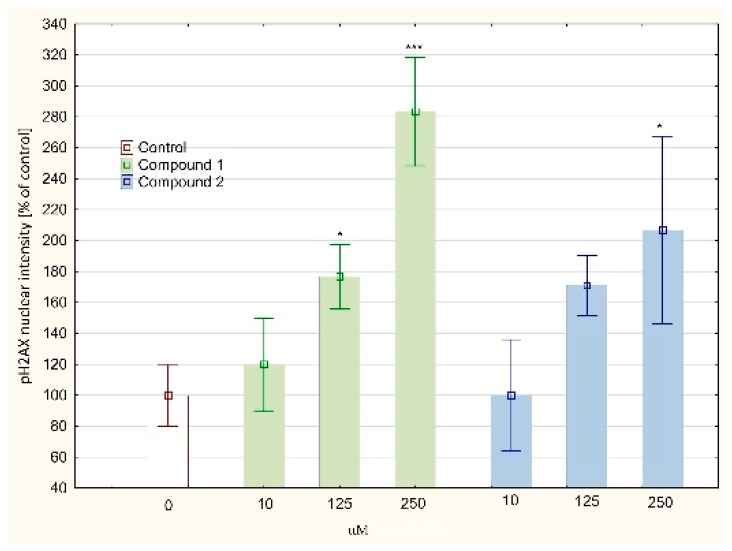
The content of double strands breaks (DSB) in tested cell’s DNA (based on phosphorylated H2AX level) presented as a % of control. The cells were treated with compound 1 or 2 for 24 h. The DSBs level was measured by specific antibody-based detection of phosphorylated H2AX (Ser139) in the nucleus. The fluorescence of secondary antibody was measured using the SpectraMax i3 Multi-Mode Platform. Values were presented as mean ± SD. * *p* < 0.05, *** *p* < 0.001 vs. control; (ANOVA followed by Tukey’s HSD post hoc analysis).

DNA synthesis disorders are closely related to DNA helix damages. The analysis of oxidative DNA damage showed a significant increase of the AP sites accumulation in the DNA isolated from the MKN74 cells treated with compound 2 in a concentration of 250 µM (12.03 ± 1.89 AP sites/100 kbp) in comparison to the control culture (1.90 ± 0.41 AP sites/100 kbp, see Figure 8). Lesser, but statistically significant increases were observed for compound 2 in concentration of 125 µM and compound **1** in a concentration of 250 µM (5.40 ± 1.21 and 4.13 ± 0.32 AP sites/100 kbp, respectively). Both tested compound treatment resulted in DNA damages in the form of DSBs, mostly compound **1** in concentration of 250 µM (283, 33 ± 35, 12% of control culture, Figure 9).

**Figure 10:**
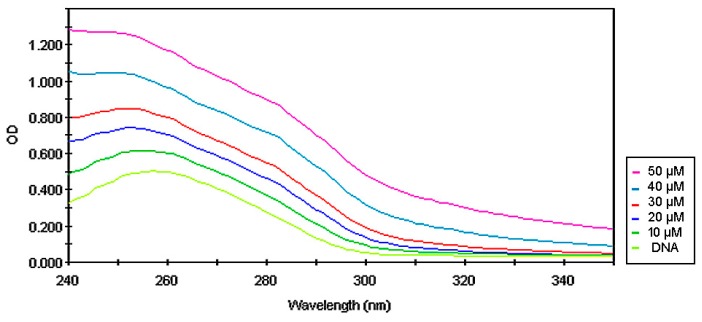
Absorption spectra of calf thymus DNA (250 µg/mL) in phosphate buffer (pH 7.4) upon addition of compound 1 (10, 20, 30, 40, and 50 µM, respectively).

**Figure 11:**
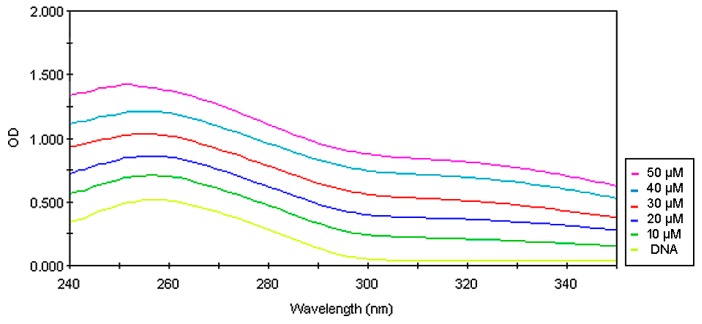
Absorption spectra of calf thymus DNA (250 µg/mL) in phosphate buffer (pH 7.4) upon addition of compound 2 (10, 20, 30, 40, and 50 µM, respectively).

Observed cell cycle arrest, as well as DNA damages, may be a result of DNA intercalation. To examine whether the tested compounds intercalate into DNA, UV–spectroscopic studies were evaluated. The spectrum of CT-DNA has been changed in the presence of varying amounts of compounds **1** and **2**. The hypochromatic effect that was observed (Figure 10 and Figure 11) generally results from the damage in the DNA double-helix structure and is connected to the intercalation of the compound [35,36].

The new thiosemicarbazide derivatives have shown toxic effects on the highly resistant gastric cancer cell line while not affecting normal cells. The selectivity of new cancer drugs is extremely important because of the serious side effects of existing therapies. The main goal of cancer therapy is to stop cell division and induce apoptosis. The tested derivatives induced apoptosis in tumor cells probably by inducing DNA damage, especially DSBs. The second of the detected DNA damages—AP sites can block the advancement of DNA replication forks, and also block the DNA polymerases [37]. For the above reasons, cell cycle arrest was expected. The analysis showed an increase in the population of cells in S phase and, additionally, in the case of compound **2** also in G2 phase. The DNA biosynthesis process involves an internal S-phase checkpoint system that limits the rate of replication in the event of physical DNA damage, especially DSB, and blocks the possibility of initiating further steps of the cell cycle by those cells. Any structural disorder forces a slowdown in the rate of replication fork motion and DNA damage response (DDR). The main goal of DDR is DNA repair or if it is ineffective, induction of apoptosis [38,39].

Based on the results obtained, it is not possible to assess the mechanism of interaction of the tested compounds on DNA. The presence of AP-sites may indicate the generation of oxidative stress [40]. In turn, UV–spectroscopic studies have shown direct interactions, i.e., the formation of complexes of tested compounds with DNA. However, the exact type of binding cannot be determined on this basis.

As experimental results described above indicated that a possible mechanism of anticancer activity of compounds **1** and **2** is interaction with DNA, we decided to use molecular docking to find how the compounds can interact with DNA. We assumed that the compounds are DNA intercalators as intercalation is one of the most common binding modes of small aromatic molecules with DNA [41]. Intercalation is the insertion of a small molecule or fragment between two adjacent base pairs (usually CG) of the DNA strand either with or without additional interactions with the grooves [33].

**Figure 12:**
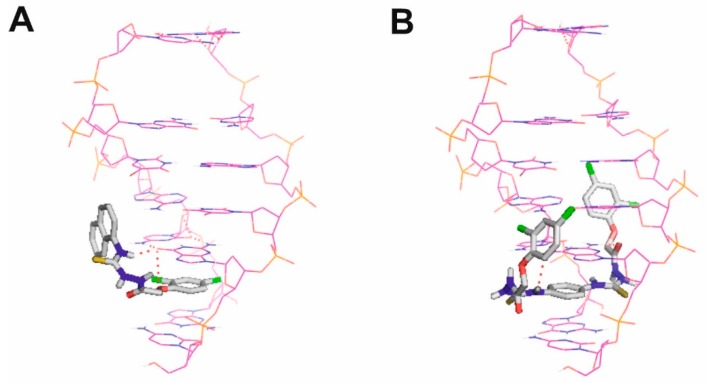
Compound 1 (**A**) and compound 2 (**B**) intercalating with DNA as obtained using molecular docking approach of flexible ligands to rigid DNA fragment. DNA is shown in wire representation with magenta carbon atoms. Ligands are shown in stick representation with grey carbon atoms. Non-polar hydrogen atoms not shown for clarity. Hydrogen bonds are shown as red dashed lines.

Figure 12 presents the docking poses of compounds **1** and **2** in a double-stranded DNA fragment. DNA intercalators commonly possess a planar polyaromatic system that forms π−π interactions with the two flanking bases [41]. These interactions are the most important driving force of the intercalation binding mode [41]. The aromatic rings of compounds **1** and **2** are located between two guanine ring systems, which constitute the intercalation binding mode. Moreover, compound **1** forms two hydrogen bonds between its 2,4-dichlorophenoxy oxygen atom and one of its nitrogen/hydrogen atom and the carbonyl group of guanine. In the case of compound **2**, a hydrogen bond is formed between one of its nitrogen/hydrogen atom and the carbonyl group of guanine, and another hydrogen bond is formed between its oxygen atom and the amino group of guanine. The values of Glide scoring functions were −6.55 for a selected pose of compound **2** and −6.48 for a selected pose of compound **1**. These values are comparable; however, they indicate slightly stronger interactions of compound **2**, which is in accordance with its better anticancer activity.

## 4. Conclusions

Compounds **1** and **2** revealed cytotoxicity against a gastric cancer cell line without significant toxicity against normal cells. Compound **1** arrested cell cycle in S and G2 phase, compound **2** visibly arrested cell cycle in S phase. Both compounds generated DNA damages in the form of AP-sites and DSBs presence. UV spectrum analysis indicates the possibility of intercalation of both compounds to DNA.

## Supplementary Materials

The following are available online at https://www.mdpi.com/2218-273X/10/2/296/s1.

## Abbreviations

AB	abasic sites
DAPI	4′,6-Diamidine-2′-phenylindole dihydrochloride
DSB	double strands breaks
MTT	Methylthiazolyldiphenyl-tetrazolium bromide
ROS	Reactive Oxygen Species
SP	Standard Precision
