# Supplementary material for: 2,4-Dichlorophenoxyacetic Thiosemicarbazides as a New Class of Compounds against Stomach Cancer Potentially Intercalating with DNA
Source: Biomolecules. 2020 Feb 13;10(2):296. doi: 10.3390/biom10020296 (PMC7072506; doi:10.3390/biom10020296)
Supplement: Supplementary file 1 [file biomolecules-10-00296-s001.pdf]

2,4-Dichlorophenoxyacetic Thiosemicarbazides as a New Class of Compounds Against Stomach Cancer Potentially Intercalating with DNA

Monika Pitucha[1*], Agnieszka Korga-Plewko[2], Pawel Kozyra[3], Magdalena Iwan[2] and Agnieszka A. Kaczor[4,5]

[1]Independent Radiopharmacy Unit, Department of Organic Chemistry, Faculty of Pharmacy, Medical University of Lublin, Poland; monika.pitucha@umlub.pl

[2]Independent Medical Biology Unit, Medical University of Lublin; agnieszkakorga@umlub.pl

[3] Student research group, Independent Radiopharmacy Unit, Medical University of Lublin,

[4]Department of Synthesis and Chemical Technology of Pharmaceutical Substances, Faculty of Pharmacy, Medical University of Lublin, e-mail: agnieszka.kaczor@umlub.pl

[5]School of Pharmacy, University of Eastern Finland, Yliopistonranta 1, P.O. Box 1627, FI-70211 Kuopio, Finland;

Compound 1

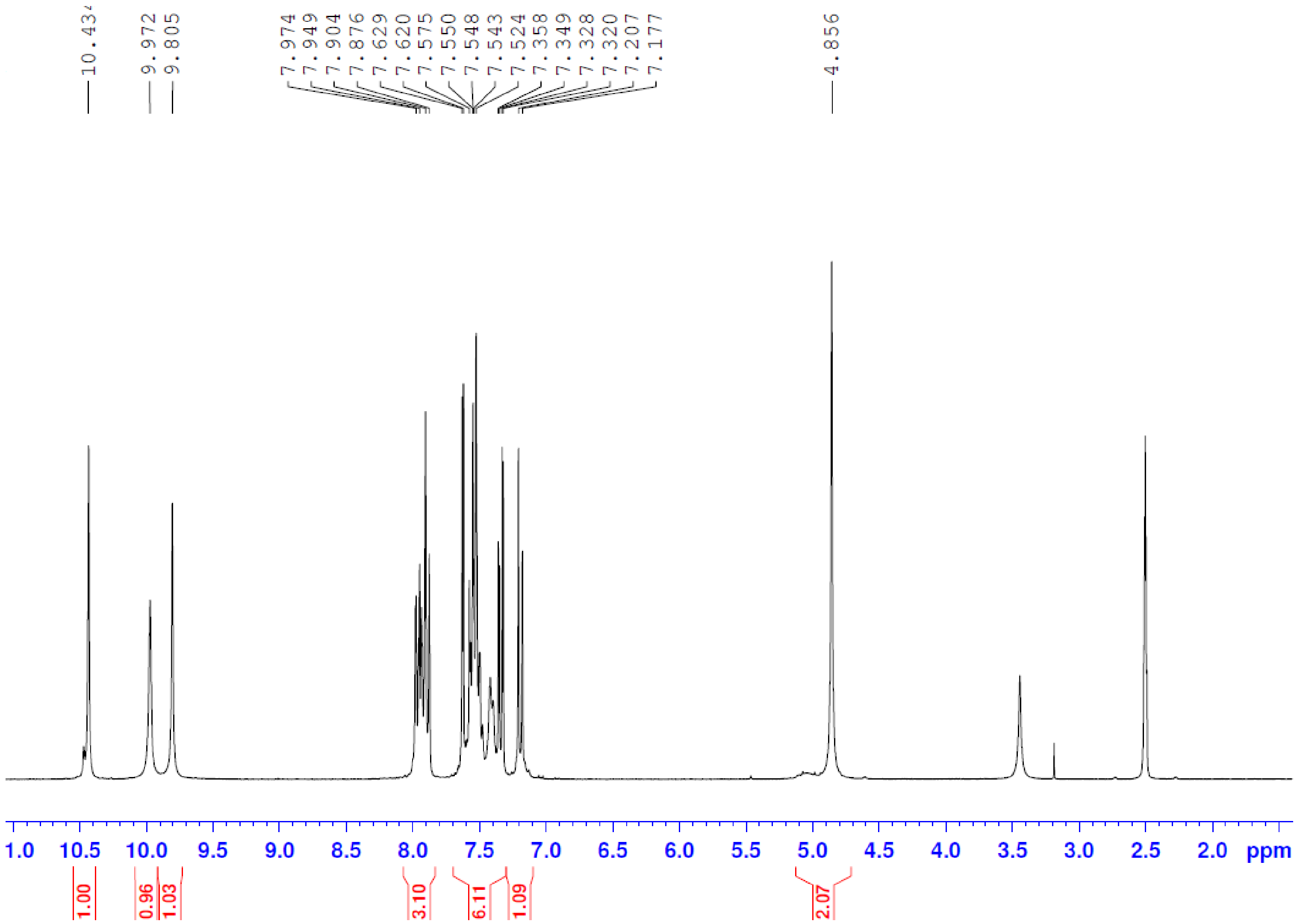

10.434
9.972
9.805
7.974
7.949
7.904
7.876
7.629
7.620
7.575
7.550
7.548
7.543
7.524
7.358
7.349
7.328
7.320
7.207
7.177
4.856
1.0 10.5 10.0 9.5 9.0 8.5 8.0 7.5 7.0 6.5 6.0 5.5 5.0 4.5 4.0 3.5 3.0 2.5 2.0 ppm
1.00
0.96
1.03
3.10
6.11
1.09
2.07

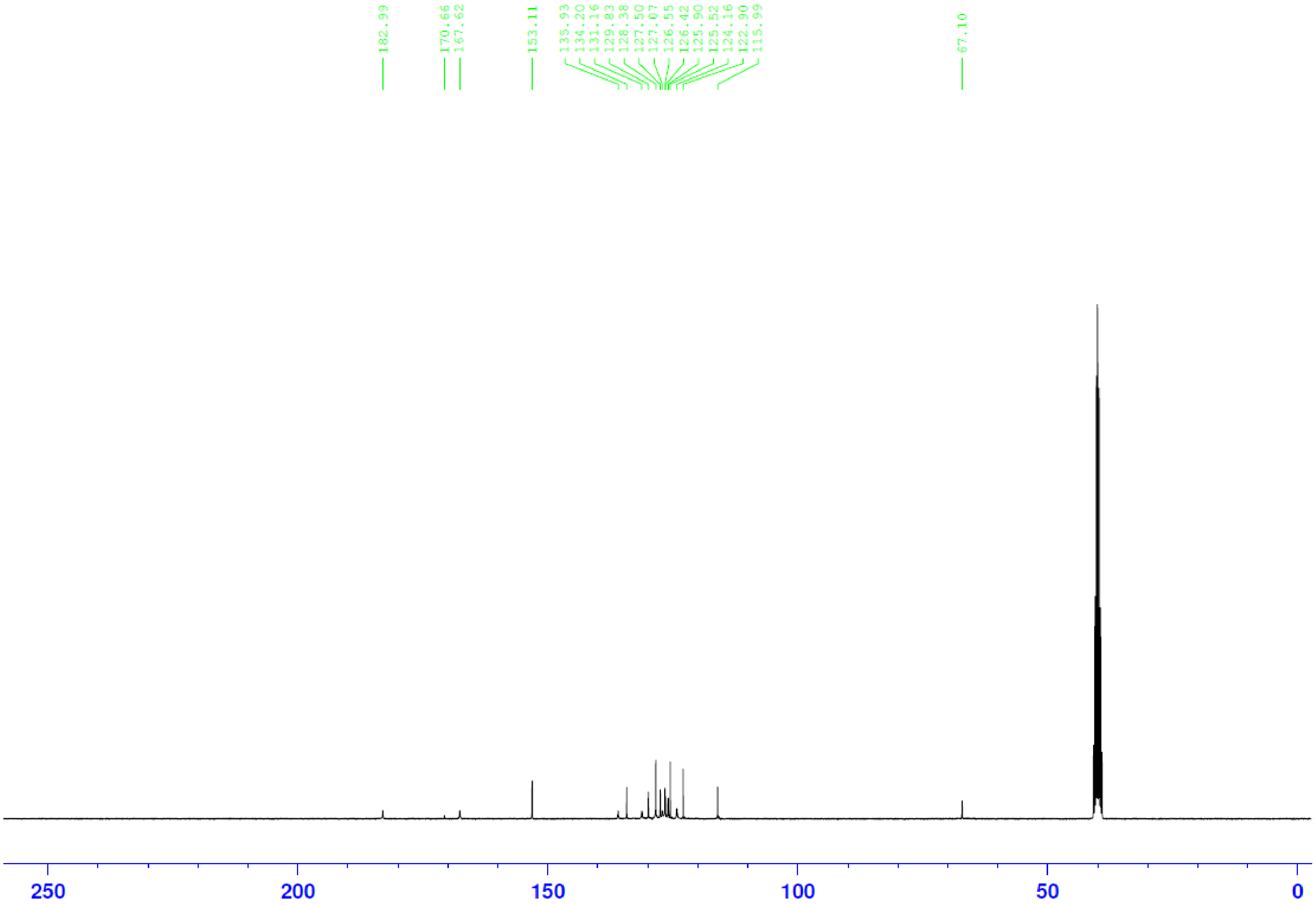
182.99
170.66
167.62
153.11
135.93
134.20
131.16
129.83
128.38
127.50
127.07
126.55
126.42
125.90
125.52
124.16
122.90
115.99
67.10
250
200
150
100
50
0

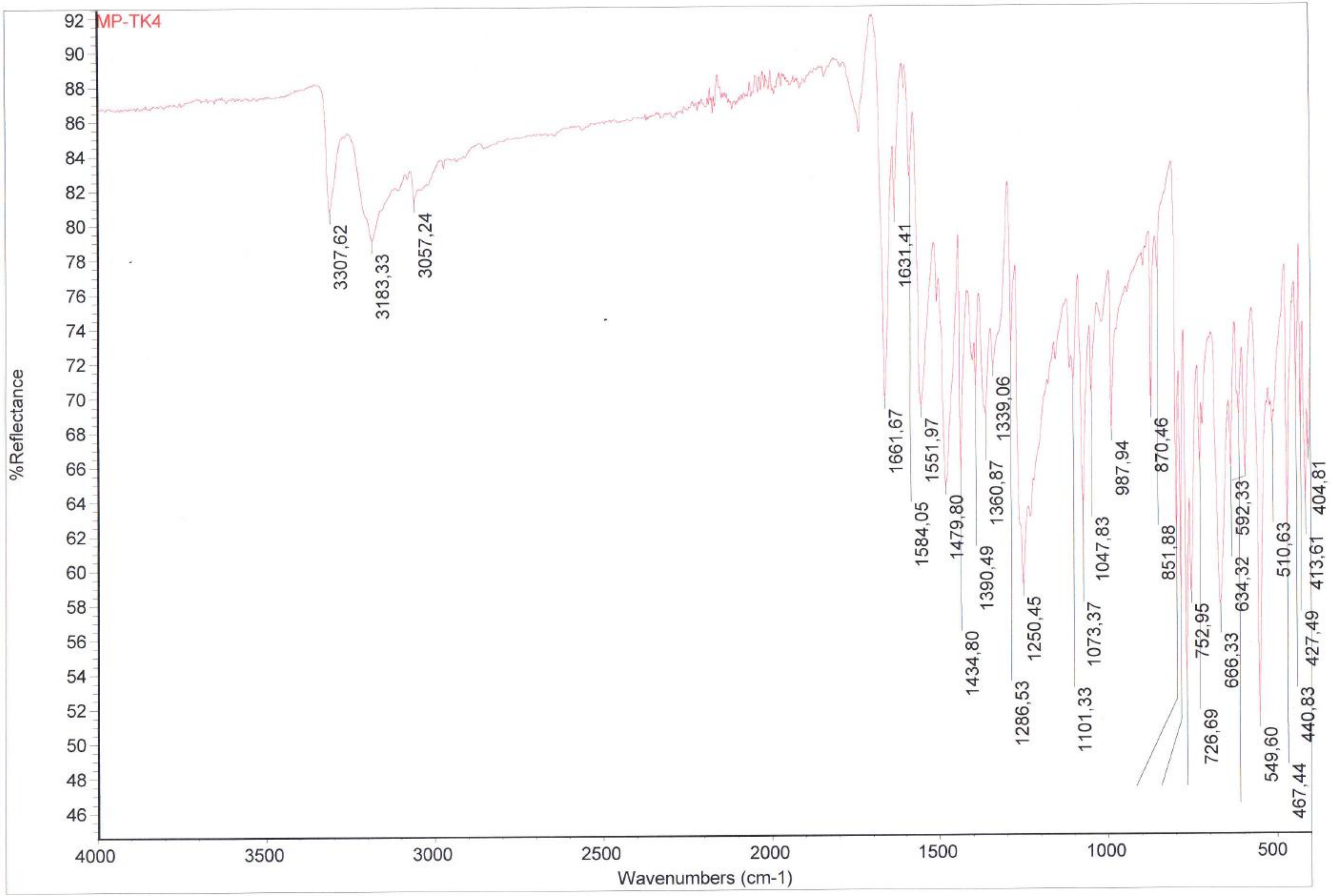
MP-TK4
%Reflectance
Wavenumbers (cm-1)
92
90
88
86
84
82
80
78
76
74
72
70
68
66
64
62
60
58
56
54
52
50
48
46
4000
3500
3000
2500
2000
1500
1000
500
3307,62
3183,33
3057,24
1661,67
1631,41
1584,05
1551,97
1479,80
1434,80
1390,49
1360,87
1339,06
1286,53
1250,45
1101,33
1073,37
1047,83
987,94
870,46
851,88
752,95
726,69
666,33
634,32
592,33
549,60
510,63
467,44
440,83
427,49
413,61
404,81

Compound 2

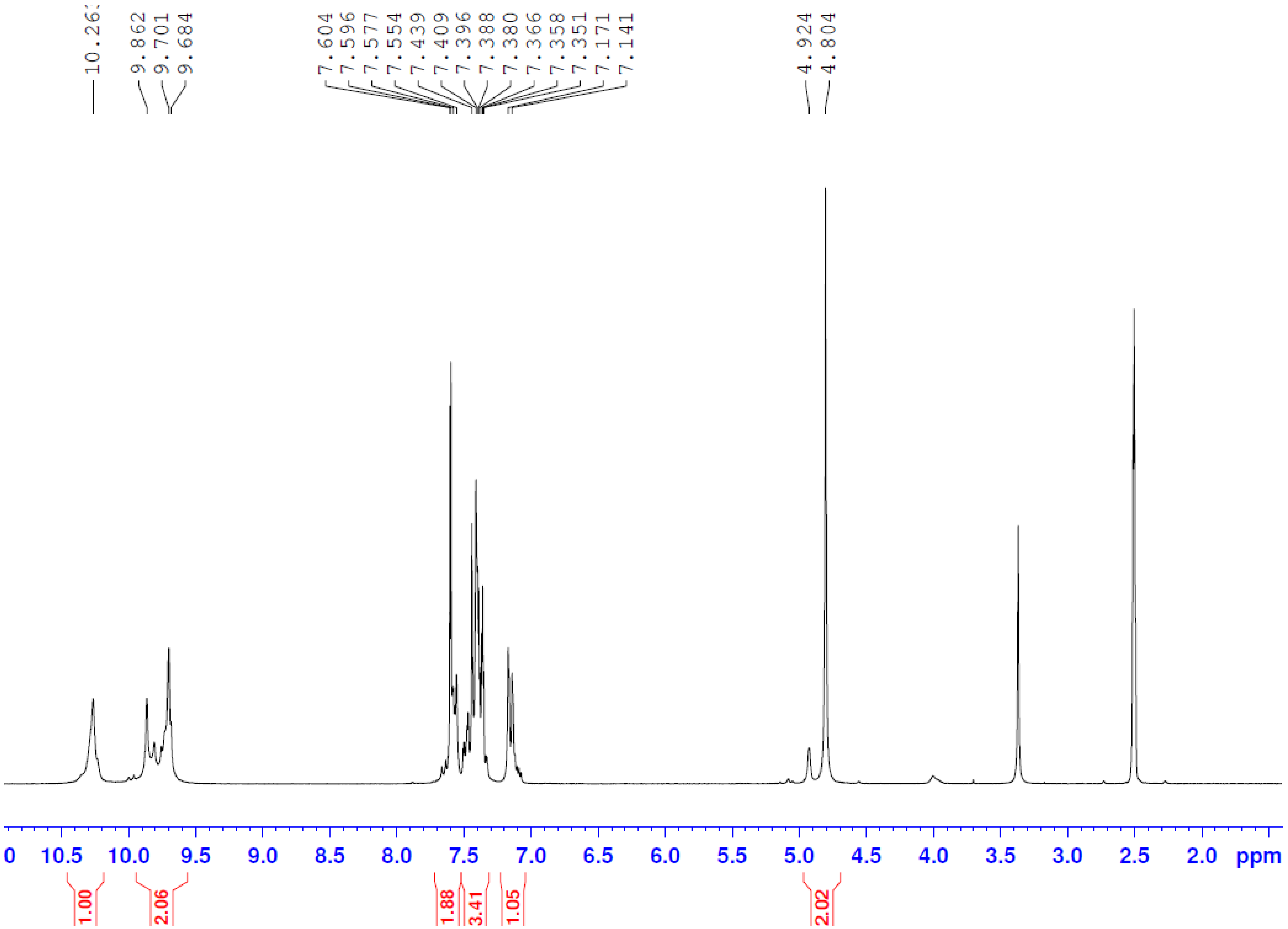
10.263
9.862
9.701
9.684
7.604
7.596
7.577
7.554
7.439
7.409
7.396
7.388
7.380
7.366
7.358
7.351
7.171
7.141
4.924
4.804
0
10.5
10.0
9.5
9.0
8.5
8.0
7.5
7.0
6.5
6.0
5.5
5.0
4.5
4.0
3.5
3.0
2.5
2.0
ppm
1.00
2.06
1.88
3.41
1.05
2.02

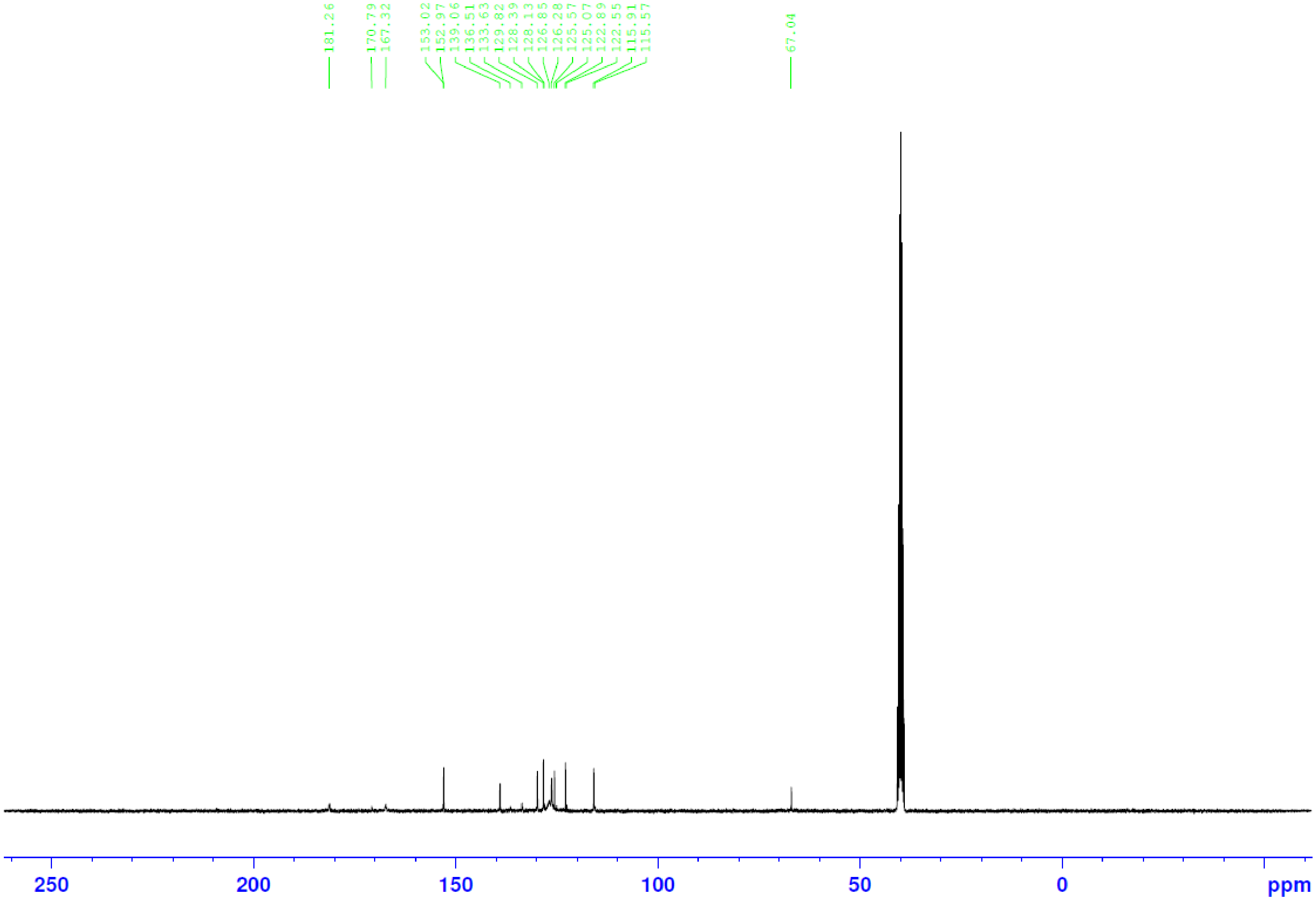

181.26
170.79
167.32
153.02
152.97
139.06
136.51
133.63
129.82
128.39
128.13
126.85
126.28
125.57
125.07
122.89
122.55
115.91
115.57
67.04
250
200
150
100
50
0
ppm

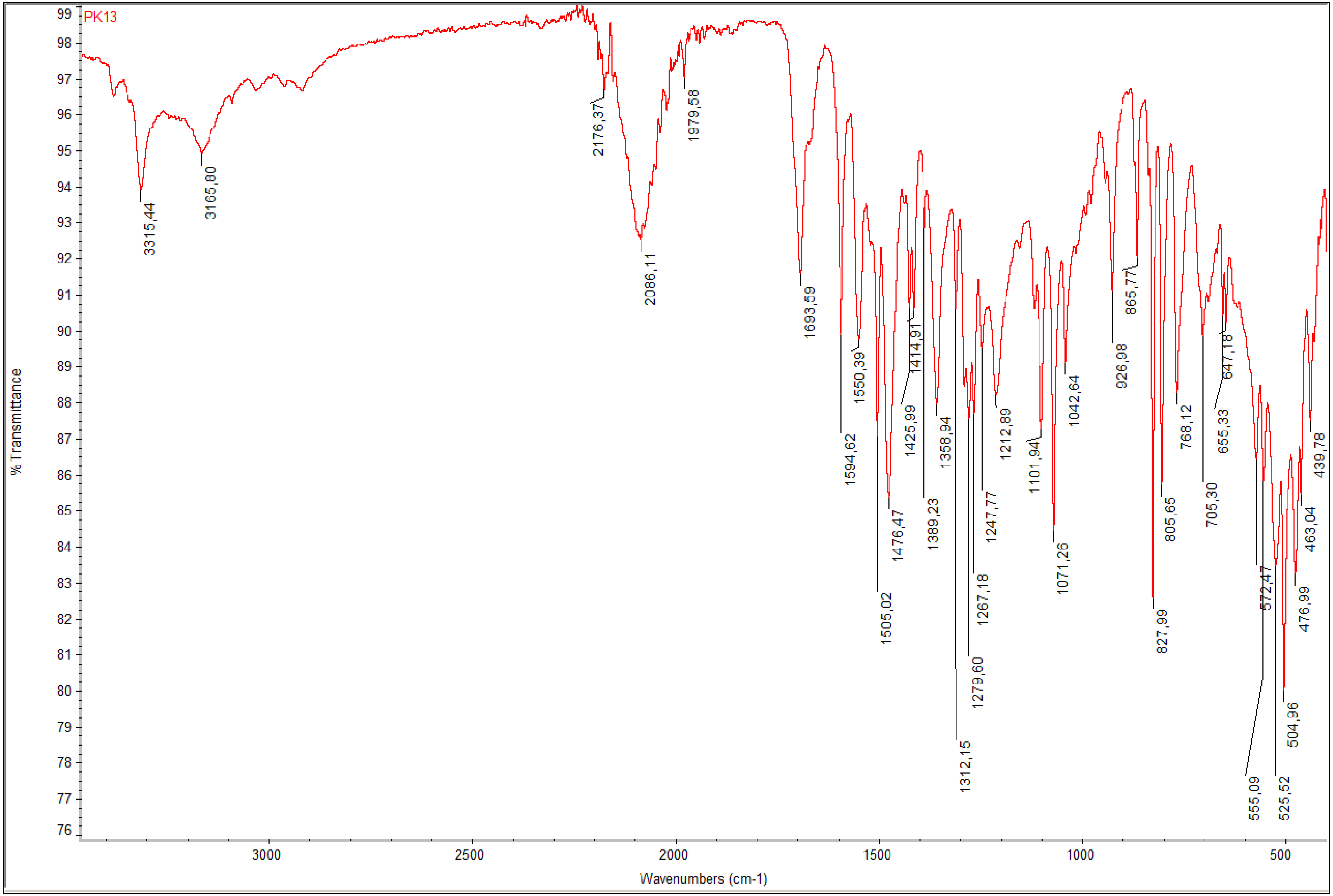

PK13
% Transmittance
Wavenumbers (cm-1)
3315,44
3165,80
2176,37
2086,11
1979,58
1693,59
1594,62
1550,39
1505,02
1476,47
1425,99
1414,91
1389,23
1358,94
1312,15
1279,60
1267,18
1247,77
1212,89
1101,94
1071,26
1042,64
926,98
865,77
827,99
805,65
768,12
705,30
655,33
647,18
572,47
555,09
525,52
504,96
476,99
463,04
439,78

Compound 1

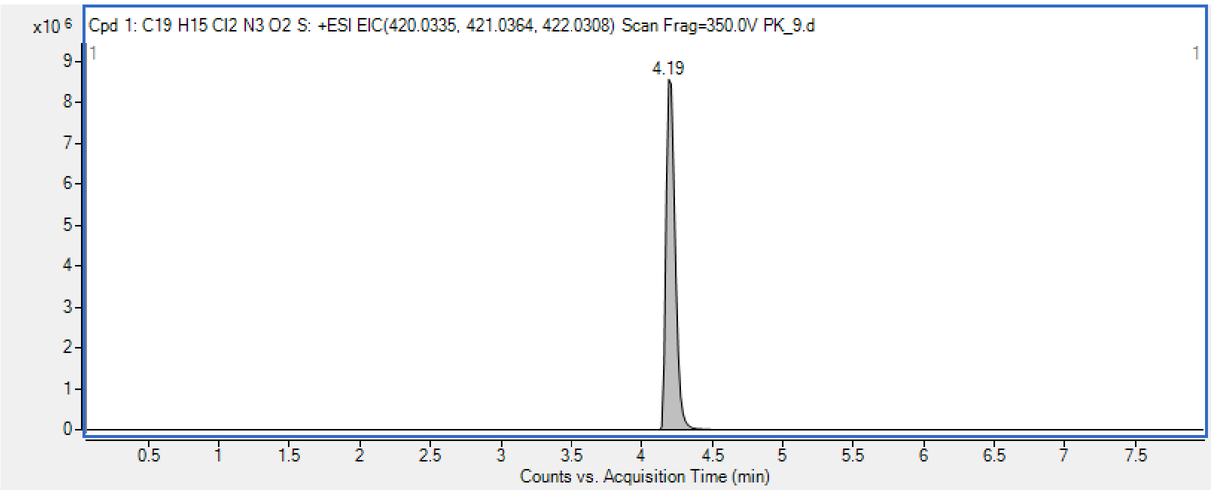

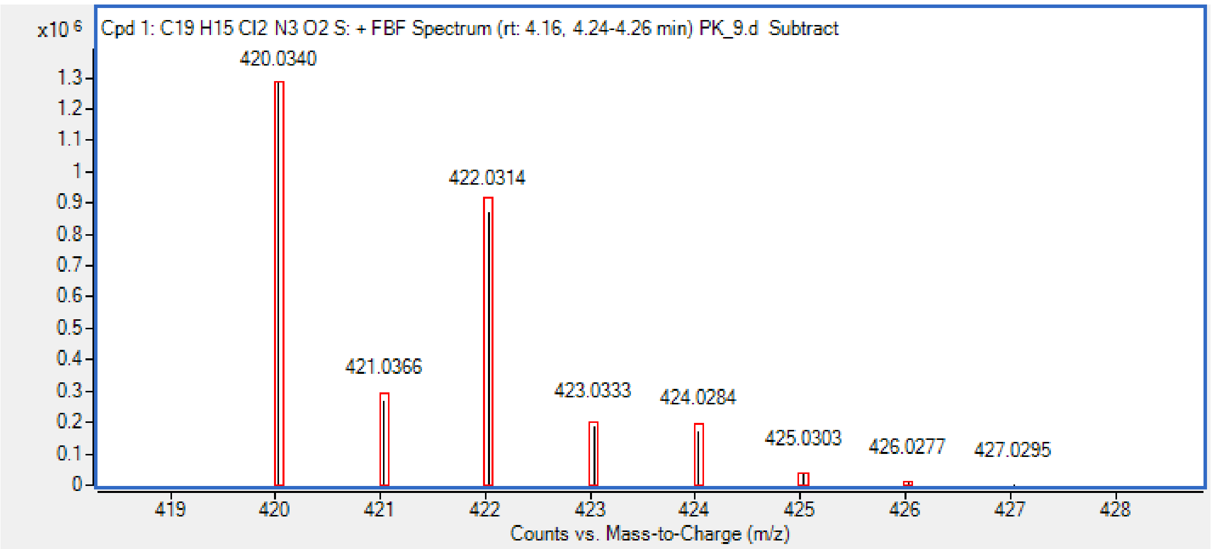

Figure S1. LC/QTOF MS EIC chromatogram (top) and MS spectrum (bottom) of compound 1. Calculated monoisotopic mass: 419.0262, Measured monoisotopic mass: 419.0266, Isotopic pattern match: 99.05%, Mass error: 0.98 ppm.

Compound 2

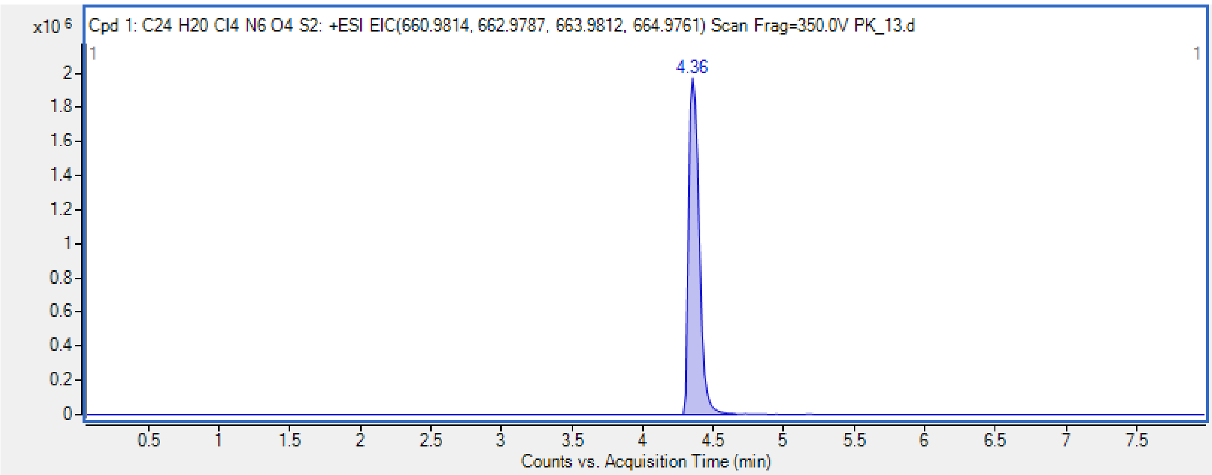

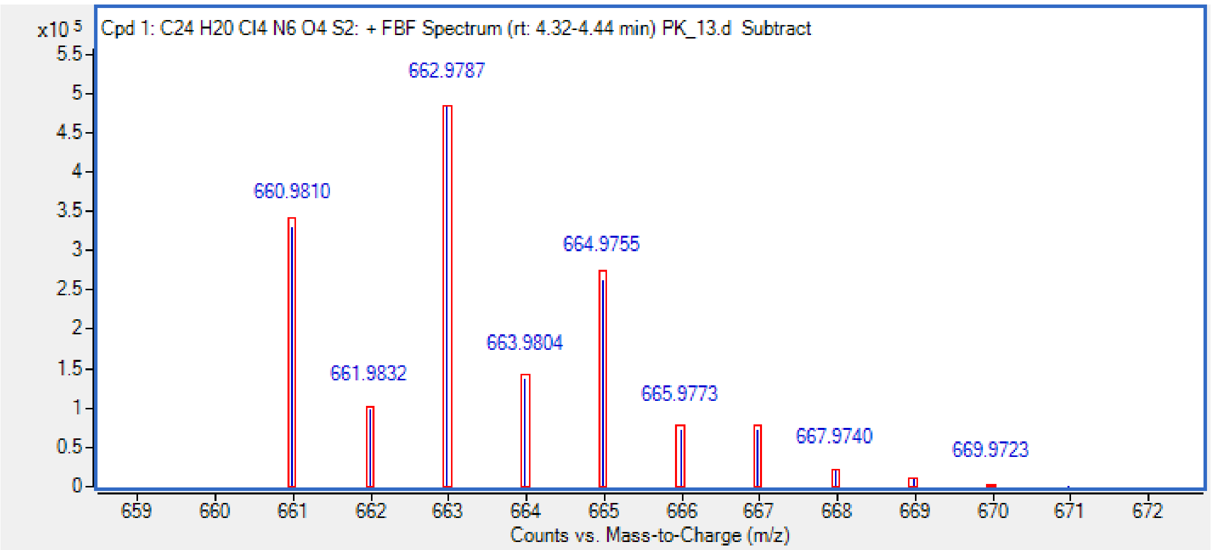

Figure S2. LC/QTOF MS EIC chromatogram (top) and MS spectrum (bottom) of compound 2. Calculated monoisotopic mass: 659.9742 , Measured monoisotopic mass: 659.9736, Isotopic pattern match: 99.33%, Mass error: -0.78 ppm.
